# Supplementary material for: Active site specificity profiling datasets of matrix metalloproteinases (MMPs) 1, 2, 3, 7, 8, 9, 12, 13 and 14
Source: Data Brief. 2016 Feb 22;7:299–310. doi: 10.1016/j.dib.2016.02.036 (PMC4777984; doi:10.1016/j.dib.2016.02.036)
Supplement: Supplementary file 10 — Supplementary material [file mmc10.zip › WebPICS_hMMP9_T_1%/P1prime.html]

 

PICS results


|  |  |
| --- | --- |
| **P1prime\_C**  5 in 138 sites   3.6 %    effects > 10 perc. pnts.  (vice-versa in brackets)  P2\_V: 32.8 (16.4)   P2\_Y: 14.9 (10.7) |  |
| **P1prime\_I**  26 in 138 sites   18.8 %    effects > 10 perc. pnts.  (vice-versa in brackets)  P1\_G: -12.1 (-14.3)   P2prime\_Q: 13.0 (24.1) |  |
| **P1prime\_M**  6 in 138 sites   4.3 %    effects > 10 perc. pnts.  (vice-versa in brackets)  P3\_A: 23.9 (11.1)   P3\_V: 40.6 (18.8)   P1\_C: 14.5 (29.0)   P1\_N: 26.1 (15.7)   P2prime\_H: 26.1 (15.7) |  |
| **P1prime\_Q**  9 in 138 sites   6.5 %    effects > 10 perc. pnts.  (vice-versa in brackets)  P2\_F: 17.9 (26.8)   P2\_H: 17.9 (26.8)   P1\_P: 13.5 (10.2)   P2prime\_C: 18.6 (33.5)   P2prime\_Q: 23.2 (14.9)   P3prime\_H: 17.9 (26.8) |  |
| **P1prime\_V**  12 in 138 sites   8.7 %    effects > 10 perc. pnts.  (vice-versa in brackets)  P2\_F: 20.7 (41.3)   P2\_M: 18.5 (24.6)   P2prime\_I: 14.1 (11.3)   P2prime\_M: 10.9 (16.3)   P3prime\_N: 11.6 (19.9) |  |
| **P1prime\_W**  3 in 138 sites   2.2 %    effects > 10 perc. pnts.  (vice-versa in brackets)  P1\_W: 31.1 (31.1)   P2prime\_M: 27.5 (10.3)   P2prime\_T: 27.5 (10.3)   P3prime\_H: 29.0 (14.5) |  |
